# The cost-saving impact of human papillomavirus self-sampling for cervical cancer screening in women workers at the manufacturing sector in Ciudad Juárez, Chihuahua, Mexico

**DOI:** 10.1371/journal.pgph.0006389

**Published:** 2026-05-04

**Authors:** Olga Georgina Martínez Montañez, Francisco Javier Picó-Guzmán, Gerardo García Zavaleta, Horacio Rafael Tinoco Vázquez, José Antonio Zamudio González, Mauricio Hernández Ávila

**Affiliations:** 1 Directorate of Economic and Social Benefits, Mexican Institute of Social Security, Mexico City, Mexico; 2 LifeSciences Consultants, Consultoría Estratégica, Mexico City, Mexico; 3 Hospital General Regional No. 2, Mexican Institute of Social Security, Ciudad Juárez, Chihuahua, Mexico; 4 Delegación Chihuahua, Mexican Institute of Social Security, Chihuahua, Chihuahua, Mexico; 5 Directorate of Economic and Social Benefits, Mexican Institute of Social Security, Mexico City, Mexico; Menzies School of Health Research: Charles Darwin University, AUSTRALIA

## Abstract

Cervical cancer is the second leading cause of cancer-related death among women in Mexico, accounting for approximately 4,500 deaths annually. The Mexican Social Security Institute (IMSS), which provides mandatory social security and healthcare for formal-sector workers, currently relies on conventional cervical cytology for early detection. To explore alternatives, a workplace-based pilot program introducing human papillomavirus (HPV) self-sampling was implemented. This study compares the effectiveness and feasibility of HPV self-sampling with the existing cytology program, aiming to inform future screening strategies. A prospective analysis of the cost-saving impact was conducted from the IMSS institutional financing perspective, including healthcare program costs and IMSS-financed disability leave and pension payments. A two-module Markov model separately simulated clinical pathways for detection, diagnosis, and treatment under each screening strategy, and the natural progression of HPV infection and cervical intraepithelial neoplasia. Model inputs were derived from the HPV self-sampling pilot and complementary literature. Costs are reported in 2024 USD and both costs and outcomes are discounted at 3% annually. Both programs were evaluated by simulating a cohort of 100,000 women over a 10 year period. The HPV self-sampling strategy was less costly and more effective than cytology. In the modeled cohort, HPV screening prevented 812 cervical cancer cases at a total cost of 39.7 million USD, resulting in a cost of 48,896 USD per cancer case prevented. In contrast, the cytology program prevented 651 cases at a cost of 99.9 million USD, yielding 153,559 USD per case prevented. HPV self-sampling demonstrated a 61% increase in the detection of high-grade cervical lesions and was less costly and more effective than cytology. The reduction in disability leaves and associated social security expenditures further amplifies its economic value for an institution like IMSS. These findings highlight the potential benefits of increasing screening coverage and implementing workplace-based HPV screening.

## Introduction

In Mexico, public and private health institutions follow a national standard for a comprehensive cervical cancer (CC) control program. This includes early detection for women aged 25–64 years every three years with conventional cytology (Pap smear) or, for women aged 35–64 years, every five years with HPV testing, plus follow-up of all abnormal or positive results for surveillance or treatment [1]. The Pap smear was the only screening test available in the national program until the partial introduction of the HPV test in 2007 in women covered by the Ministry of Health (without social security) [2].

Although the national CC program has resulted in significant reductions in both incidence and mortality in Mexico, cervical cancer remains the second leading cause of cancer death among women. In 2023, it caused 4,500 deaths, with a mortality rate of 6.3 and an incidence of 13.2 per 100,000 (world age-standardized rate) [3,4]. The incidence rate is nearly three times higher than the target set by the World Health Organization for its elimination. This shortfall is largely due to opportunistic screening, inadequate program implementation stemming from insufficient funding and a high prevalence of other concurrent health issues. The situation has been further exacerbated by the onset of the COVID-19 pandemic, highlighting the need for enhanced prevention and early detection efforts [2,5].

The Mexican Social Security Institute (IMSS) is a government agency that provides social security services to private-sector employees and their families. These services include preventive, primary and specialized healthcare without copayments, as well as pensions, retirement benefits, disability insurance and occupational risk coverage. Funded through employer contributions, employee payroll deductions, and government support, IMSS covers approximately 22.3 million workers —42.1% of whom are women— and extends benefits to nearly 63 million individuals. Notably, 70% of insured female workers are between the ages of 30 and 69, making them a high-priority group for cervical cancer prevention [6].

In 2023, IMSS reported performing 4,125,294 cervical cytology tests, representing 29.4% of affiliated women aged 25 years and older [7]. HPV testing is currently not implemented within IMSS, which may limit access to high-performance cervical cancer screening for working women covered by the institution. Additionally, women in the workforce often face time constraints that reduce their ability to attend preventive services at medical units [8]. Global evidence indicates that screening with the HPV test is more effective and cost-efficient than traditional cervical cytology [9]. Moreover, HPV self-sampling methods improve access to screening, as women report preferring this method due to its comfort, privacy, and ease of use compared to cervical cytology [10–12]. In recent years, improved tests for HPV screening have been introduced. However, common challenges for effective implementation persist, including updating standards and guidelines, strengthening follow-up and treatment capacity, and developing a comprehensive quality assurance plan [13–15].

To improve cervical cancer screening access for female workers aged 35–65, IMSS launched a pilot program introducing HPV self-sampling in manufacturing workplaces in Ciudad Juarez, Chihuahua. The initiative assessed the feasibility of integrating this strategy into the IMSS framework and was based on a collaboration among government, employers, and employees [16].

The pilot project was conducted in collaboration with the National Council of the Mexican Export Manufacturing Maquiladora Industry, which represents 6,600 companies employing 3.3 million workers, 37.7% of whom are women, all insured by IMSS. The pilot took place in Ciudad Juárez, Chihuahua, from February 2023 to March 2024. The workforce included approximately 212,000 women, with 108,998 of them aged 35–64. Of these, 74,855 were employed in the manufacturing industry, which comprised 321 companies with more than 50 workers [17]. IMSS invited 145 representatives from the maquiladora industry, primarily health personnel, to participate in a pilot program and attend a three-day training course. The participating health personnel received comprehensive training on implementation requirements, printed pamphlets and posters, sample collection supplies, and guidance on organizing informational meetings. They were also instructed on providing designated times for workers to participate, setting up appropriate spaces for self-sampling, registering women, and coordinating the transportation of samples to IMSS facilities. Ninety-four factories (64.8%) accepted the invitation and completed the course, while 65 factories (44.8%) actively engaged in the program activities [16]. Building on the data generated by this pilot, the present study analyzes the effectiveness and cost-saving impact of the HPV self-sampling approach compared to cervical cytology. It highlights the potential public health impact of workplace-based screening strategies for public insurers such as IMSS.

## Materials and methods

### Ethics statement

The evaluation of the pilot program was approved by the Research and Ethics Committee of the Mexican Social Security Institute under registration number R-2023-785-060. This analysis is a secondary use of data from that evaluation and, therefore, did not require additional ethics approval.

### Context and population

This study is a cost-saving analysis based on a two-module dynamic Markov model. Clinical inputs, epidemiological parameters, and cost data were derived primarily from the pilot workplace-based HPV self-sampling program implemented among female manufacturing workers in Ciudad Juárez, Mexico, and were complemented by a targeted literature review.

Details of the recruitment procedures, participation rates, and comparative performance of HPV testing versus Pap smear coverage, as well as clinical follow-up outcomes from the pilot program, have been published elsewhere [16]. Data management and analysis were conducted in accordance with national standards ensuring confidentiality [18].

All women aged 35–64 years employed in the participating factories were eligible for HPV self-sampling, except those who were pregnant or had undergone a hysterectomy for benign causes. No incentives were offered for participation other than the potential health benefits. From a target population of 19,847 eligible women, 8,539 (43.0%) verbally agreed to participate and performed HPV self-sampling between February 2023 and February 2024. HPV self-sampling results were integrated into the IMSS cervical cancer screening registry.

For comparison, cytology (Pap smear) records of female workers aged 35–64 years residing in Ciudad Juárez were also analyzed. The assessment of precursor lesions and invasive cervical cancer excluded women with prior cervical cancer, a history of high-grade precursor lesions, or current cervical cancer symptoms, to focus on asymptomatic screening populations.

### Data sources

The IMSS screening registry includes HPV results, cytology reports, and, when results are positive or abnormal, colposcopy evaluations, biopsy findings, and subsequent case management information. Follow-up, treatment, and screening outcomes were supplemented by using electronic medical records from IMSS. Additionally, cytology and biopsy records from the three IMSS hospitals in Ciudad Juarez were extracted and cross-matched with the screening database to minimize under-registration. All data used for this analysis was obtained between November 1st, 2024, and March 21st, 2025.

### Study perspective

The cost-saving analysis was conducted from the institutional financing perspective of the IMSS, considering it as a tripartite social insurance and the main provider of contributory healthcare for formal-sector workers and their dependents in Mexico. The evaluation included direct programmatic costs such as screening logistics, HPV testing and cytology procedures, diagnostic confirmation through colposcopy and biopsy, outpatient management of precancerous lesions, and treatment of cervical cancer across all stages.

Costs associated with temporary disability leaves and pensions linked to cervical cancer were also included, as they represent direct financial obligations for IMSS. Indirect and intangible costs, such as patient transportation, caregiver time outside formal employment, and quality-of-life metrics, were not considered.

### Interventions

Two cervical cancer screening strategies were compared: 1) From the current conventional cervical cytology, worker women aged 35–64 years were selected from the screening registries, the cytology was classified according to the Bethesda System. Abnormal results included atypical glandular cells (AGC), atypical squamous cells of undetermined significance (ASC-US), atypical squamous cells - cannot exclude high-grade squamous intraepithelial lesion (ASC-H), low-grade squamous intraepithelial lesion (LSIL), high-grade squamous intraepithelial lesion (HSIL), and malignant neoplasm. 2) HPV self-sampling strategy (workplace-based pilot program). Self-collected vaginal samples were analyzed using the cobas® HPV test detecting 14 high-risk HPV types, including HPV-16, HPV-18, and a pooled group of 12 hrHPV (high-risk HPV) types (31, 33, 35, 39, 45, 51, 52, 56, 58, 59, 66, 68) [19]. Women testing positive for HPV-16/18 were referred directly to colposcopy, whereas women positive for other hrHPV types underwent triage cytology. After a positive result, the woman received an email with guidance on follow up at IMSS facilities. Follow up, diagnostic work-up, and treatment were provided in the same IMSS healthcare units and services as for women with abnormal Pap smear results. Colposcopy and histological lesions were classified using the cervical intraepithelial neoplasia (CIN) system, where CIN1 corresponds to LSIL, and CIN2, CIN3, and carcinoma in situ correspond to HSIL.

**Fig 1:**
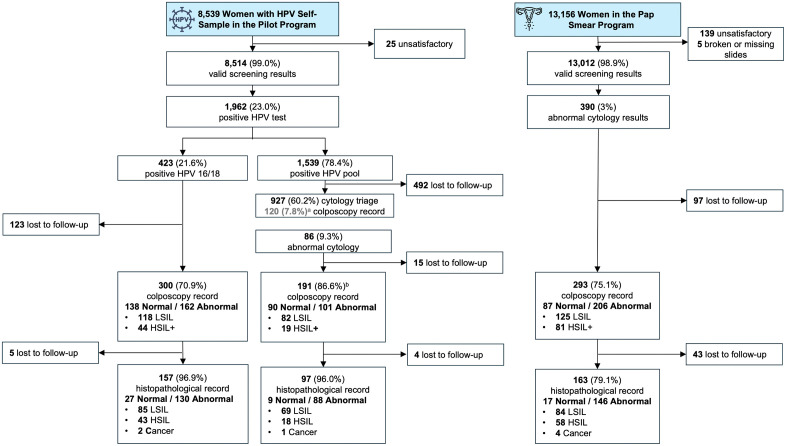
Flowchart of screening outcomes and diagnostic follow-up in the HPV self-sampling and Pap smear programs.

Among 19,847 eligible workers, 8,539 women (43.0%) participated in the HPV self-sampling program, yielding 8,514 valid samples. Of these, 1,962 women (23.0%) tested positive for HPV, including 423 (21.6%) with HPV16/18 and 1,539 (78.4%) with other high-risk HPV types. A total of 61 women were diagnosed with HSIL and 3 with invasive cervical cancers, while 639 (32.6%) were lost to follow-up. In the Pap smear program, adherence to colposcopy among women with abnormal cytology was 75.1%, and adherence to histopathological follow-up was 79.1%. Overall, 58 women were diagnosed with HSIL and 4 with invasive cancer, and 140 women (35.9%) were lost to follow-up. The screening workflows and corresponding population outcomes for each program are presented in Fig 1 and Tables 1 and 2.

**Table 1:** HSIL and cancer detection per 1,000 screened with Pap smear and HPV self-sampling tests.

Age-specific	Pap smear *n* = 13,012					HPV self-sampling *n* = 8,514				
	Tests	HSILs		Cancer		Tests	HSILs		Cancer	
**Cases**	**Rate**	**Cases**	**Rate**	**Cases**	**Rate**	**Cases**	**Rate**
35–39	2,379	17	7.15	0	–	1,706	21	12.31	1	0.
40–44	2,746	12	4.37	1	0.36	2,100	23	10.95	0	–
45–49	2,902	13	4.48	0	–	1,963	7	3.57	1	0.51
50–54	2,633	9	3.42	0	–	1,605	7	4.36	1	0.62
55–59	1,776	6	3.38	3	1.69	948	3	3.16	0	–
60–64	576	1	1.74	0	–	192	0	–	0	–
**Total**	**13,012**	**58**	**4.46**	**4**	**0.31**	**8,514**	**61**	**7.16**	**3**	**0.35**

**Table 2:** Characteristics of women screened by Pap smear and HPV self-sampling, comparing age and screening history.

Screened women	Pap smear	HPV self-sampling
Mean age (95% CI)	47.09 (46.97, 47.21)	46.11 (45.97, 46.26)
Number of women and percent		
35–44 years old	5,125 (39.3%)	3,806 (44.7%)
45–64 years old	7,887 (60.7%)	4,708 (55.3%)
Number and percent of women without a Pap smear registered previously		
35–44 years old	1,572 (30.7%)	3,014 (79.2%)
45–64 years old	2,507 (31.7%)	3,601 (76.6%)

The figure summarizes the flow of women participating in the pilot HPV self-sampling program (left) and the conventional Pap smear program (right) conducted in Ciudad Juarez. ^a^ 120 women had colposcopy record without cytology triage in the HPV+ Pool positive group. ^b^ The percentage is only for women with abnormal results in cytology triage with colposcopy follow-up. HSIL+ = high-grade squamous intraepithelial lesion or worse.

### Study design

**Fig 2:**
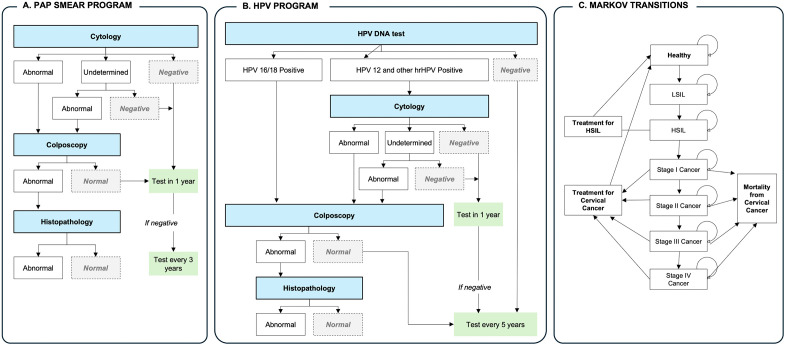
Diagnostic pathways for cytology and HPV screening, including triage steps, diagnosis, management, and the Markov transitions used in the cost-saving model.

This study evaluated the cost-saving impact of alternative cervical cancer screening strategies using a de novo, two-module cohort state-transition (Markov) model. The model simulated transitions between population screening pathways and the natural history of HPV infection and cervical carcinogenesis. The two-module structure was chosen to separately represent: (1) the clinical pathways of detection, diagnosis, and treatment under cytology and HPV-based screening, capturing the impact of each strategy on resource use (HPV tests, cytology, colposcopy) and the distribution of precursor lesions and invasive cancers detected at different stages; and (2) the population-level progression of cervical intraepithelial neoplasia (CIN) and cervical cancer, estimating the total number of invasive cases and their associated costs, including treatment expenditures and IMSS social security payments (temporary disability and pensions). The overall transition structure is depicted in Fig 2.

The Markov model simulates cervical cancer screening and disease progression over time by screening an initial cohort of 100,000 women in the first year and then allowing transitions between health states to represent their potential clinical outcomes across subsequent years. Average life expectancy for women in Mexico (78 years) was used to approximate the remaining lifetime over which cervical cancer-relative costs accrue beyond the 10-year screening period.

The time horizon was selected to adequately capture the medium-term natural history of HPV infection and the downstream clinical and economic impact of both screening strategies. Under the HPV self-sampling program, HPV-negative women are re-screened every five years, resulting in an average of two screening cycles per woman within the model period. Under the Pap smear program, women with abnormal results are followed annually and cytology-negative women are re-screened every three years, yielding an average of three to four screening cycles per woman over ten years.

For each strategy, total costs were calculated by adding screening program costs, management costs for HSILs, treatment costs for cervical cancer by stage, and IMSS social security payments due to cervical cancer-related disability and death. Per 100,000 women, the model incorporates an annual incidence of 19.5, one-year prevalence of 21.8, and cumulative five-year prevalence of 91.7 [20,21]. Mortality rates were calculated based on the last 10 years of data from the affiliated population of Ciudad Juárez (17.0 per 100,000 women) [20,22]. Positivity rates and follow-up data for cytology and HPV programs were based on the pilot study, and sensitivity, cancer staging, and treatment effectiveness metrics were informed by previous studies, as presented in Table 3. Medical management for all confirmed cervical cancer cases follows the clinical guidelines published by IMSS [29], and the treatment costs used are derived from previously published analyses that applied these same institutional guidelines. All calculations, including resource use and cost-saving outputs, were implemented using Microsoft Excel and R.

**Table 3:** Model parameters and data sources for test performance, epidemiological indicators, and treatment effectiveness used to compare Pap smear and HPV self-sampling strategies.

Variable	Value
•Test sensitivity at different stages	
LSILs [23,24]	Pap Smear: 45% HPV Testing: 71%
HSILs [23,25,26]	Pap Smear: 59% HPV Testing: 98%
•Positivity rates per 1000 women^a^	
HSILs	Pap Smear: 4.46 HPV Testing: 7.16
Cancer	Pap Smear: 0.31 HPV Testing: 0.35
•Follow-up rate^a^ (percentage)	Pap Smear: 75.1% HPV 16/18: 70.9% HPV Pool: 60.2%
•Sensitivity analysis (follow-up rate)	90% for both programs
•Abnormal cytology after positive HPV test^a^	HPV Testing: 9.3%
•Annual incidence of CC in Ciudad Juárez with current program [7]	19.5 per 100,000 women
•Mortality rate of CC in Ciudad Juárez (10-year average) [22]	17.0 per 100,000 women
•Prevalence of cervical cancer [21]	
1 year	21.8 per 100,000 women
5 years	91.7 per 100,000 women
•Precancerous lesion grades 5-year distribution [27]	
CIN1	83%
CIN2	6.9%
CIN3	9.2%
•Effectiveness of treatments [28]	
I	88%
IIA	88%
IIB	64%
III	64%
IV	44%
•Transition from CIN 1–2/3 [28]	4%
•Transition from CIN 2/3 to FIGO Stage I [28]	10%
•Transition from FIGO Stage I to II [28]	15%
•Transition from FIGO Stage II to III [28]	36%
•Transition from FIGO Stage III to IV [28]	21%

^a^HPV Self-sampling Pilot.

### Estimated resources spent and costs

All costs were standardized to December 2024 values to ensure comparability across data sources from 2022 to 2024. Cost inputs originally reported in Mexican pesos (MXN) were inflated using the official INEGI National Consumer Price Index (INPC) [30] as follows: Cost_Dec2024(MXN) = Cost_t(MXN) × (INPC_Dec2024/ INPC_t), where t corresponds to the year (or month, when available) of the original cost estimate. When the exact month of costing was not available, we assumed costs were expressed in average prices for that year and used the annual average INPC as INPC_t. Inflated costs were then converted to U.S. dollars (USD) using the exchange rate as of December 2024, which equaled 20.05 Mexican pesos (MXN), as reported by Banco de México. The analysis followed current CHEERS recommendations for reporting economic evaluations. Costs and outcomes were discounted at 3% per year in the base-case analysis, consistent with standard economic evaluation practice over multi-year horizons. Sensitivity analyses examined alternative annual discount rates of 0% and 5%. No formal willingness-to-pay (WTP) threshold was defined, as this evaluation is intended to inform IMSS internal budget prioritization rather than external cost-effectiveness benchmarking. The institutional objective is to maximize the number of cervical cancer cases prevented and to optimize allocation of limited resources.

### Clinical costs

All costs represent direct institutional costs for IMSS from the provider perspective and are not reimbursement tariffs or charge rates; they reflect the estimated economic cost of delivering service. Unit costs for cytology and HPV testing were obtained from the IMSS’s Department of Budgeting and Finances, using standardized annual costings that include personnel, consumables, laboratory operations, and infrastructure. These unit costs are based on IMSS internal microcosting estimates that allocate total annual expenditures (e.g., salaries, supplies, laboratory operations, utilities, maintenance, and other overhead) across procedures using standardized costing methods and service volumes, thereby reflecting provider economic costs rather than billed amounts. The total cost per cytology test was 24.52 USD per woman screened. This estimate encompasses nurse and clinical staff time for sample collection, patient support, and specimen handling; test reagents and consumables (slides, collection brushes, fixatives); laboratory operations (technical and administrative staff, utilities, and maintenance); and infrastructure costs associated with dedicated clinical space and equipment for sample collection and processing.

In contrast, the total cost per HPV test was 17.71 USD per woman screened. This cost includes reduced nurse and staff time due to self-sampling; HPV test reagents and processing materials (collection devices, transport media, pipettes, cartridges, and other laboratory consumables); and laboratory operational costs (staff, indirect expenses, and equipment use). Infrastructure costs for sample collection are not included for HPV self-sampling, as the collection occurs outside the clinical setting and does not require dedicated examination rooms or clinical staff presence during the procedure.

Although the unit reagent cost of the HPV test is higher than that of cytology (14.87 vs 9.37 USD per test, respectively), the overall efficiency gains —measured in terms of nursing throughput (70 HPV self-sampling kits vs. 26 cytology procedures per session) and laboratory processing capacity (658 HPV samples vs. 50 cytology slides per day, according to IMSS standards)— result in lower total screening costs per woman for the HPV self-sampling strategy. The reported nursing throughput and laboratory processing capacities reflect IMSS operational standards used as planning assumptions for resource and budget estimation and represent average observed throughput at individual facilities during the pilot period. Colposcopy and histopathology costs were also provided by the Department of Budgeting and Finances, including infrastructure, consumables, and professional interpretation. Costs for the managing of high-grade squamous intraepithelial lesions (HSILs) and treatment of cervical cancer were derived from the most recent (2017) IMSS Diagnostic-Related Groups report [31,32].

### Disability leaves and social costs

The model considered the broader social impact of cervical cancer across four dimensions frequently highlighted in the literature for middle-income settings: caregiver productivity loss, presenteeism, disability leaves, and paid pensions [33]. Caregiver productivity loss refers to reduced work performance or work time of a family member or close contact due to caregiving responsibilities, while presenteeism captures reduced on-the-job productivity among patients who remain employed despite disease-related symptoms. Evidence from countries comparable to Mexico indicates that productivity losses account for a substantial share of the indirect economic burden of cervical cancer [33–35].

In line with the IMSS institutional financing perspective, disability leaves and pensions—both of which represent direct financial obligations for IMSS—were monetized and included in the base-case cost-savings analysis. Estimates of caregiver productivity loss and presenteeism were used to contextualize the broader socioeconomic burden of cervical cancer and to inform scenario analyses but were not incorporated into the main resource-use and cost outcomes.

IMSS covers temporary disability payments at 60% of the worker’s registered salary, starting on the fourth day of disability and extending up to 52 weeks, with a potential extension of up to 26 additional weeks based on IMSS medical evaluations. Using data from the most recent IMSS disability leave report and the IMSS Subsidies Datamart 2025 from the Unit of Economic Benefits, we estimated an average of 79 subsidized days per cervical cancer case, with a mean daily disability payment of 18.59 USD.

Paid pensions were estimated based on annual cervical cancer–related deaths and the reported distribution of pension types (widowhood, orphanhood, and ascendancy) in the latest IMSS report [6]. For each death, pension types were assigned according to these proportions and calculated the expected present value of IMSS pension payments. For ascendancy pensions, a single annual payment was assumed due to their low frequency among total cases. For orphanhood pensions, an average parental age of 21.3 years at the child’s birth was assumed, with pension payments continuing until the child reached 18 years of age [36,37]. Widowhood pensions were modeled assuming age parity between spouses and were paid until the surviving spouse reached a life expectancy of 75 years. Table 4 summarizes these social security cost components and their parameter values.

**Table 4:** Clinical and socioeconomic parameters incorporated into the cost-saving model, including IMSS unit costs, salary and disability metrics, subsidy data, and additional economic inputs.

Resource	Cost USD
***Clinical Costs***	
^•^Cost per HPV test^a^	$17.71
Nurse time	12%
Test	84%
Laboratory and supplies	3%
^•^Costs for colposcopy in HPV program^a^	$108.77
•Cost per cytology^a^	$24.52
Nurse time	16%
Supplies	4%
Test	38%
Laboratory and supplies	21%
Infrastructure for sample collection	20%
•Costs for colposcopy and histological follow-up	$114.82
_•_Average cost of treatment per stage [32]	
HSIL	$2,040.05
IA–IB	$7,394.42
IIA–IIB	$6,226.06
IIIA–IIIB	$8,893.76
IVA–IVB	$10,426.22
***Disability Leaves and Social Costs***	
•Annual base salary^b^	$9,562.82
•Days subsidized per case^b^	79 days
•Average payment per subsidized day^b^	$18.59
•Caregiver productivity loss [35]	
I	29.7%
II	32.4%
III	32.9%
IV	40.5%
•Days with presenteeism [6]	
I	95
II	95
III	78
IV	None
•Pensions [6]	
Widowhood (%) and yearly cost	50.6%; $19,356.21
Orphanhood (%) and yearly cost	31.5%; $4,647.98
Ascendancy (%) and yearly cost	3.5%; $2,776.46

a According to the Cost of Services 2022 report by the IMSS Department of Budgeting and Finances.

b According to the IMSS Subsidies Datamart 2025 from the Unit of Economic Benefits.

c All costs are presented in USD as of December 2024, which equaled 20.05 MXN.

### Effectiveness measures

The primary economic outcome was the cost per cervical cancer case prevented. For each strategy, an average cost per case prevented was calculated by dividing the total costs of the program (including screening, diagnosis, treatment, and IMSS social security payments) by the number of cervical cancer cases averted compared with no screening. Incremental results were assessed by computing the incremental cost per cervical cancer case prevented, defined as the difference in total costs between strategies divided by the difference in the number of cases prevented over the 10-year horizon.

### Sensitivity analysis

#### Deterministic sensitivity analysis.

A deterministic one-way sensitivity analysis was conducted to assess the robustness of the results to uncertainty in key model parameters. The analysis focused primarily on follow-up adherence among women with positive screening results, as this is a critical driver of both clinical effectiveness and costs. In line with the World Health Organization Cervical Cancer Elimination Initiative, which recommends that at least 90% of women with a positive high-risk HPV test complete appropriate follow-up (e.g., colposcopy, repeat testing, or treatment) [38], follow-up rates were varied from those observed in the pilot program up to this 90% benchmark. The impact of improved follow-up on the number of cervical cancer cases prevented and the cost per case prevented was evaluated for each screening strategy.

Additional, one-way deterministic sensitivity analyses were performed on other influential parameters, including HPV test unit cost, HPV positivity, colposcopy referral rates and unit costs, and treatment costs for HSIL (CIN2–3) and cervical cancer. Each parameter was varied individually across plausible ranges while all other inputs remained at base-case values. Incremental cost was defined as the difference in total costs between the HPV self-sampling strategy and cytology (HPV minus cytology).

A threshold (break-even) analysis was also conducted for the HPV test unit cost, identifying the price at which the HPV self-sampling strategy ceased to be cost-saving (incremental cost = 0), while all other parameters remained fixed.

Results from the one-way sensitivity analyses were summarized using a tornado diagram, which displays the change in incremental total cost relative to the base-case estimate when each parameter was set to its lower or upper bound. Negative deviations indicate greater cost savings for HPV self-sampling, whereas positive deviations indicate reduced cost savings. Parameters were ranked by the magnitude of their impact on incremental cost.

#### Probabilistic sensitivity analysis.

To account for joint uncertainty across multiple parameters, a probabilistic sensitivity analysis (PSA) was conducted using Monte Carlo simulation with 10,000 iterations. In each iteration, uncertain parameters were simultaneously sampled from predefined probability distributions and propagated through the Excel-based model. This generated a distribution of incremental costs and the probability that HPV self-sampling was cost-saving (incremental cost < 0).

Follow-up adherence parameters were modeled using beta distributions (bounded between 0 and 1), centered on observed pilot data (Pap smear: 75.1%; HPV16/18: 70.9%; pooled high-risk HPV: 60.2%). Where denominators were available, beta distribution parameters were derived using the binomial conjugate approach (α = successes + 1; β = failures + 1), based on the number of HPV16/18-positive women (n = 423) and pooled high-risk HPV-positive women (n = 1,539).

Unit costs for screening tests (HPV and cytology) were modeled using lognormal distributions to ensure positivity and to reflect uncertainty around IMSS micro-costing estimates. Base-case costs were taken from the IMSS costing report and expressed in 2024 USD (HPV test: $17.71; cytology: $24.52). PSA results are reported as the mean and 2.5th–97.5th percentiles (95% uncertainty interval) of incremental costs, as well as the probability that HPV self-sampling is cost-saving Table 5.

**Table 5:** Parameters included in the probabilistic sensitivity analysis (PSA).

Parameter	Base-case value	Distribution	Data source
Follow-up rate after HPV positive (HPV16/18 → colposcopy)	0.709	Beta	*Observed follow-up rate and HPV16/18 positives from IMSS pilot/program data*
Follow-up rate after HPV positive (pooled hrHPV → cytology triage/ follow-up)	0.602	Beta	*Observed follow-up rate and pooled hrHPV positives from IMSS pilot/program data*
Follow-up rate after abnormal Pap smear (colposcopy adherence)	0.751	Beta	*Adherence reported for Pap smear program; registry contains colposcopy/biopsy follow-up fields*
Unit cost per HPV test	$17.71	Lognormal	*IMSS costing estimate for HPV test cost*
Unit cost per cytology (Pap smear)	$24.52	Lognormal	*IMSS costing estimate for cytology cost*

## Results

### Cost and outcomes

The HPV screening program was less costly and prevented more cervical cancer cases (dominant) than the traditional Pap smear program. The prospective model projected that the HPV screening program with a 70.9% of follow-up rate for HPV 16/18 and 60.2% for HPV Pool would incur a total cost of 39.7 million USD, potentially preventing up to 812 cases of cervical cancer over ten years. The traditional program with a 75.1% follow-up was estimated to cost 99.9 million USD and prevent 651 cases of cervical cancer. The cost per case prevented was calculated at 48,896 USD for the HPV program compared to 153,559 USD for the Pap smear program, indicating that the HPV screening costs 68% less per case prevented. In a secondary analysis from the health services perspective (excluding IMSS-financed disability leave and pension payments), total clinical costs were 11.9 million for HPV self-sampling and 20.3 million USD for cytology, corresponding to 14,955 and 31,125 USD per cervical cancer case prevented, respectively. Results were robust to discounting assumptions. Using 0% and 5% annual discount rates for both costs and outcomes did not change the conclusion that HPV self-sampling remained less costly and prevented more cases than cytology (dominant). Table 6 presents these results.

**Table 6:** Total program costs and outcomes for Pap smear and HPV self-sampling, including clinical and social expenditures, cervical cancer cases prevented, and cost per case prevented from the total and health services perspectives.

Strategy	Health Services Cost^a^	Disabilities and Social Cost^a^	Total Cost^a^	Cervical cancer cases prevented	Cost per case prevented (Total)	Cost per case prevented (Health services)
Pap Smear	20.3	79.7	99.9	651	$153,559	$31,125
HPV	11.9	27.8	39.7	812	$48,896	$14,955

^a^All costs ($) in Millions US Dollars 2024.

Projecting the model’s results to the Maquiladora Industry population alone would prevent approximately 4,500 cervical cancer cases nationwide and generate savings of up to 274 million USD, justifying the investment in women’s health.

### Disability leaves and social impact

**Fig 3:**
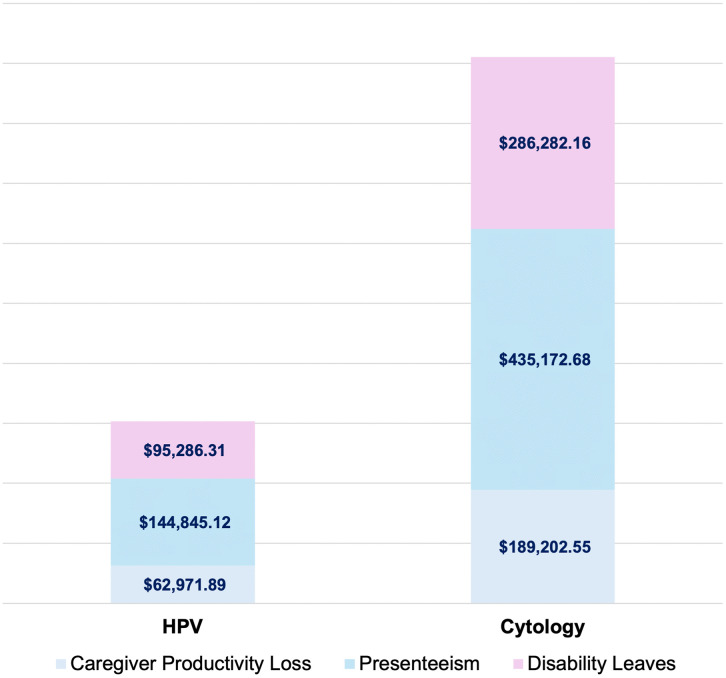
Social cost components for HPV self-sampling and Pap smear screening —excluding pensions— covering sick leave, productivity losses, and caregiver-related expenses.

The current Pap smear program represents a total social impact of 79,663,106 USD; the HPV self-sampling program incurs a social cost of 27,765,512 USD, reflecting a 65% reduction. Of the total social impact, caregiver productivity loss accounts for 0.2%, presenteeism for 0.5%, disability leaves for 0.4%, and pensions for 98.9%. Pensions represent the largest share of total expenses, as they are calculated over extended periods, either until children reach working age in the case of orphanhood, or up to a life expectancy of 75 years for widowhood. Because the HPV program results in fewer deaths (60 vs 177), its associated pension expenditure is lower, totaling 27.5 million compared to 79.1 million USD under the current Pap smear program. Fig 3, shows the breakdown of social costs for each program, excluding pension-related expenses.

#### Disability days.

**Fig 4:**
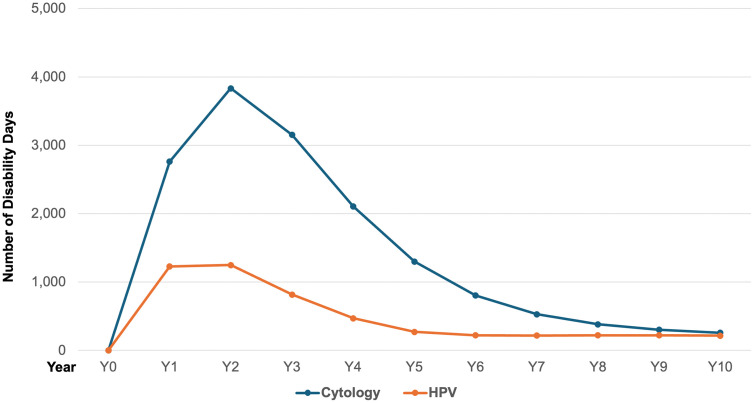
Annual disability days paid by IMSS under Pap smear and HPV self-sampling.

This study highlights the importance of disability leave costs, as caregiver productivity loss and presenteeism represent indirect economic impacts of cervical cancer, while disability leaves and pensions are direct expenses borne by the institution. Fig 4, illustrates the annual number of disability days associated with each program, showing a decrease as cervical cancer cases are reduced through each strategy. Disability leave is also a burden for women and their families, who reduce household income by 45% during general illness and affects a socioeconomic group that might not be able to cover basic needs [34].

The Pap smear program results in 15,399 disability days, costing the institution 286,282 USD. In contrast, the HPV program reduces disability days to 5,125 (-67%), with a projected expense of 95,286 USD. The savings from the HPV program are further underscored by its impact on reducing disability leave costs by 67%, which translates to substantial savings for IMSS. Higher lesion detection rates within the HPV group also demonstrate the program’s effectiveness in identifying precancerous conditions early, leading to better health outcomes and decreased treatment costs. Incorporating both clinical and social costs, the HPV program delivers a total savings of 60%, with an overall cost of 39.7 million compared to the current strategy’s 99.9 million USD.

### Sensitivity analysis

Although the program has demonstrated a 68% lower cost per case prevented at current follow-up rates, achieving higher adherence is essential for the success of cervical cancer screening. This analysis examines the potential outcomes if follow-up rates increase to 90% for both HPV and Pap programs.

**Fig 5:**
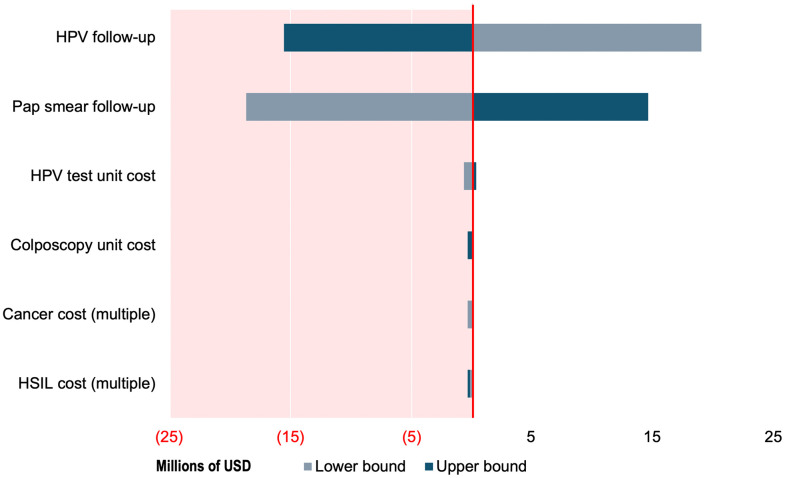
One-way sensitivity analysis (tornado) for incremental total cost (HPV self-sampling − cytology).

In one-way sensitivity analysis, the incremental total cost (HPV − cytology) was most sensitive to follow-up completion parameters, particularly follow-up after a positive HPV test and follow-up after cytology. The unit cost of HPV testing was also an important driver, while uncertainty in colposcopy unit costs and treatment cost parameters for HSIL and cervical cancer produced comparatively smaller changes within the ranges evaluated. Overall, results indicate that the economic conclusions are primarily driven by screening pathway completion and HPV testing costs. Fig 5, summarizes the main drivers of incremental costs.

**Fig 6:**
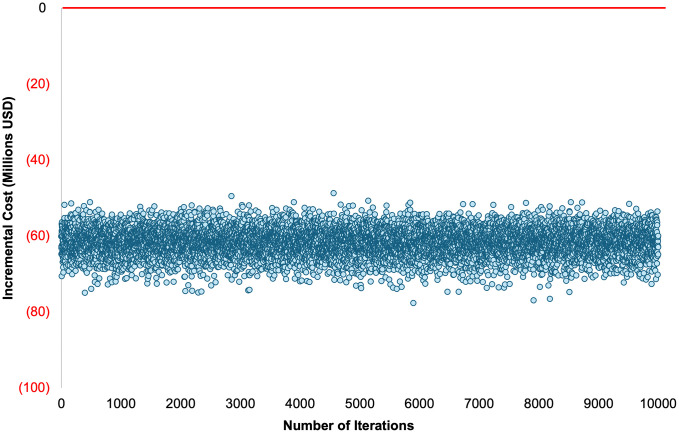
Probabilistic sensitivity analysis. Scatter plot of incremental costs from 10,000 Monte Carlo simulations comparing HPV screening with the current program.

In the base case, HPV self-sampling remained cost-saving until the HPV test price reached 262.7 USD per test. Probabilistic sensitivity analysis showed that the HPV screening strategy was cost-saving in 100% of simulations. Fig 6, illustrates the distribution of incremental costs across 10,000 Monte Carlo iterations, with all values remaining below zero. The mean incremental cost was 61.7 million USD, with a 95% uncertainty interval (2.5th–97.5th percentiles) from 69.1 to 55.0 million USD.

In the HPV program, this would prevent an additional 49 cervical cancer cases per 100,000 women over ten years (+6%), or 743 cases prevented compared to 592 with the current follow-up rate. The HSIL detection rate would increase from 8.3 to 10.3 per 100,000 women, a 23% increase in early detection. The cost per case prevented would decrease from 167,818.46 to 52,998.14 USD (-68%), improving the cost per case prevented.

## Discussion

Women play a crucial role as part of the workforce and in sustaining the economies of their communities and households. This study examines the economic impact of cancer on women, considering not only medical care but also its effects in the workplace, on the economic benefits to which they are entitled, and, to a lesser extent, within their homes.

Prospective modeling suggests that the HPV self-sampling program could prevent up to 812 cancer cases over 10 years, exceeding the 651 cases preventable by the Pap smear program. Additionally, the HPV screening program not only incurred lower total costs but also demonstrated a reduced cost per case prevented. These projections underscore the economic and public health rationale for prioritizing HPV screening as the main preventive approach in Mexico.

Furthermore, the social impact of an HPV testing program is evidenced by significant savings in disability leave payments (-67%) resulting from timely detection, highlighting the economic benefits. This represents not only substantial cost savings for IMSS as a social security institution but also potential financial benefits for participating companies. As more women undergo screening, cancer incidence decreases, thereby reducing productivity losses and enhancing potential savings for enterprises. This dual benefit underscores the importance of collaborative efforts between public health institutions and the private sector in cervical cancer prevention.

When comparing both programs at higher follow-up rates, the HPV program remains superior, with a greater reduction in cervical cancer cases and 76% lower cost per case prevented. While the absolute cost levels observed in our analysis may differ from those reported in other settings, cross-study comparisons should be interpreted cautiously because published evaluations often use different effectiveness outcomes (e.g., cost per QALY, years of life saved, cost per CIN2 + detected, or discounted lifetime costs) rather than cost per cervical cancer case prevented. Threshold and deterministic sensitivity analyses suggest that the conclusion of cost-savings is robust: HPV self-sampling remains cost-saving across wide variations in key parameters and only ceases to be cost-saving at substantially higher HPV test prices.

Several international economic evaluations have compared HPV-based screening with cytology under settings with high follow-up (≥90%), generally reporting favorable economic profiles for HPV screening, although using effectiveness metrics that differ from the natural-unit outcome applied in our analysis. For example, a study in Switzerland reported a lower cost per QALY for HPV versus Pap smear (12,413 vs 22,488 USD) [39], while an analysis in China reported cost per year of life saved estimates that favored cytology over HPV (577 vs 959 USD) [40]. Other studies have used alternative outcomes such as cost per CIN2 + detected (e.g., Sweden: 5,261 vs 17,454 USD) [41] or discounted lifetime cost estimates (e.g., Australia: 57 vs 80 USD) [42]. Because these studies report different effectiveness endpoints (QALY, YLS, CIN2 + detection, or lifetime costs) than the cost per cervical cancer case prevented used here, they are not directly comparable “one-to-one”; therefore, they are included only as contextual evidence that HPV screening may offer economic advantages across a range of settings, with differences driven by outcome definitions, modeling assumptions, time horizons, follow-up adherence, and included cost components.

Comparable studies conducted across 89 low- and middle-income countries have reported HPV test costs averaging 9.12 USD, largely due to preferential pricing agreements established with national governments. However, at the time of this analysis, no evidence of such negotiated pricing between IMSS and HPV test providers was available [43].

Our findings support HPV self-sampling as a highly sensitive strategy that is dominant (lower costs and greater impact) than cytology in this analysis. This approach aligns with global evidence identifying it as one of the most effective interventions for cervical cancer prevention [41,44–46]. Modeling results indicate that achieving 70% coverage can reduce cervical cancer incidence by 50% or more within five years; our study observed a 61% reduction, reinforcing its effectiveness [42]. Based on these results, implementing HPV self-sampling in Mexico’s female workforce could accelerate cervical cancer elimination, particularly among women who have never been screened, due to its convenience and acceptability [47–50].

The cost savings and improved health outcomes associated with the program advocate for policy changes and increased public health investment in HPV screening and follow-up protocols. Furthermore, these results underscore the importance of enhancing follow-up adherence to maximize the benefits of such screening programs with more health promotion and education and informatics tools for women and health personnel [2,8]. To reduce costs and manage the increased number of women requiring triage and higher colposcopy referral rates in HPV-based screening, several interventions have been proposed, these include HPV extended genotyping, RNA-based testing, and methylation marker analysis, which also reduce the follow-up loss associated with Pap of triage [51,52]. For example, prioritizing HPV16/18-positive cases for colposcopy while using additional molecular markers has been shown to reduce unnecessary referrals by up to 95% without compromising lesion detection [40,53]. Similarly, RNA-based testing for E6/E7 mRNA improves specificity, leading to a 63% reduction in colposcopies. Integrating these approaches into screening programs can improve the cost per case prevented, reduce unnecessary procedures, and minimize patient distress while maintaining diagnostic accuracy [41].

Future research and implementation efforts should prioritize sufficient investing in strategies for the accelerated elimination of cervical cancer including offering vaccination to women attending screening just as the FASTER concept [8,54], update norms and guidelines, strengthen diagnosis and treatment capacity, and develop a comprehensive quality assurance plan for the screening process [2,46,55,56].

We identified the following limitations of this analysis. First, the study does not include the operational costs associated with follow-up activities, such as phone calls, reminders, and administrative coordination, which may lead to a slight underestimation of the total program cost. Second, treatment cost estimates were sourced from a previously published IMSS study (2019). While these data adhere to national clinical guidelines, they may not fully reflect the costs of newly introduced treatments or recent updates in therapeutic practice.

The effectiveness of screening is influenced by program coverage, registry accuracy, adherence to follow-up protocols, quality assurance in colposcopy and histopathology, and disease prevalence among the target population. In this analysis, both HPV self-sampling and conventional cytology encounter comparable limitations, including incomplete case registration, quality control, and suboptimal follow-up compliance.

## Conclusions

Implementing HPV self-sampling within workplace health programs represents a transformative opportunity for cervical cancer prevention in Mexico. The combined evidence from the pilot and the prospective modeling shows that HPV screening is not only more sensitive than cytology but also demonstrates a cost-saving impact with lower costs and greater effectiveness, and socially advantageous by reducing productivity losses and healthcare expenditures. While challenges remain in ensuring follow-up adherence and optimizing triage, the findings provide a clear policy pathway toward scaling HPV-based screening nationwide. Strengthening collaboration between public institutions like IMSS and private employers can further enhance women’s access to preventive care, moving Mexico closer to the World Health Organization’s goal of cervical cancer elimination.

## Supporting information

S1 Data Population data sets. **Sheet 1**: Cytology program. Includes the following variables: ID, Age, Cytology date, Cytology post HPV, Cytology result, Colposcopy date, Colposcopy post HPV, Colposcopy result, Biopsy date, Biopsy post HPV, Biopsy result. **Sheet 2:** HPV program. Includes the same variable structure, corresponding to participants enrolled in the HPV self-sampling program.
(XLSX)

S1 Table Modeling inputs used for the Markov model. (XLSX)
